# Experimental and Theoretical Investigations of the Constitutive Relations of Artificial Frozen Silty Clay

**DOI:** 10.3390/ma12193159

**Published:** 2019-09-27

**Authors:** Zhiming Li, Jian Chen, Chaojun Mao

**Affiliations:** 1 School of Civil Engineering, Harbin Institute of Technology, Heilongjiang, Harbin 150090, China; lizhiming1117@163.com; 2 Department of Civil and Environmental Engineering, Nagaoka University of Technology, Nagaoka, Niigata 940-2188, Japan; 3 Third Engineering Company Limited of the First Highway Engineering Bureau of China Communications Construction Company, Beijing 100000, China

**Keywords:** artificial frozen silty clay, constitutive relation, statistical damage, triaxial experiment

## Abstract

The strength and deformation characteristics of artificial frozen soils are quite sensitive to temperature, confining pressure, and water content. To investigate these effects, a series of triaxial compressive tests on frozen Harbin silty clay were conducted at temperatures of −5 °C, −10 °C, and −15 °C under different confining pressures and water contents. From the stress–strain curves under lower water content and confining pressure, strain–softening behavior was observed. The modified Duncan–Chang (MDC) model was employed to describe the constitutive relations of artificial frozen silty clay while considering the strain–softening effects. After introducing statistical damage (SD) theory, an SD constitutive model with the failure strain as a random variable was proposed, which is able to overcome the drawbacks of the MDC model. The predicted SD model results are found to be consistent with the experimental results.

## 1. Introduction

Artificial ground freezing (AGF), which is widely applied in tunnel construction, coal mining, foundations, and other underground construction, is an economical and effective method with the advantages of suitability, non–pollution, waterproofness [1,2,3,4]. Studies on the strength and deformation characteristics of artificial frozen soils have been a crucial research focus because of the complicated performance of frozen soils, which is significantly influenced by temperature, confining pressure, and water content. Therefore, various experiments have been conducted to reveal the mechanical properties of frozen soils [5,6,7,8,9,10]; likewise, considerable effort has been made toward understanding the relationship between load and deformation in constitutive models of frozen soils. Within the framework of continuum mechanics, a series of constitutive models, such as nonlinear elastic, elasto-plastic, and viscoelastic–plastic models, were proposed by different researchers from different viewpoints. Beginning in the early 1930s, Tsytovich [11] pioneered the exploration of the constitutive relations of frozen sand at various stress rates and temperatures through a series of uniaxial experiments. Later, additional competing constitutive models based on experimental results or theoretical analyses were proposed [12,13,14,15,16,17,18,19,20].

Frozen soil is a special soil–water system because of the presence of ice inclusions, which cause it (especially its mechanical properties) to be highly susceptible to temperature, confining pressure, and water content. The influences of these factors are not independent, but are coupled with each other [21]. Therefore, the effect of these factors on the strength of frozen soil has been widely studied by scholars [22,23,24,25,26]. Wu [27] pointed out that there exists a peak strength value of frozen soil for varying moisture content. The strength of frozen soil continues to increase until the water content approaches saturation, then decreases with a further increase in water content and tends toward ice strength. Wu [28] found that the relation between uniaxial compressive strength and temperature of frozen soil was a power function. Ma [29] conducted a series of triaxial experiments and explained that the reason why strength of frozen soil decreases with confining pressure was that ice melting happened at stress concentration points. Although there are plenty of research achievements regarding the strength of frozen soil, almost all research is limited to a single or double factor analysis.

The Duncan–Chang (DC) model, which has several parameters with definite physical meaning, is widely applied in theoretical calculation and engineering guidance. However, strain–softening of frozen soil can be observed under lower water content and confining pressure when deformed beyond a certain limit, which cannot be described effectively by a nonlinear elastic DC model. Much work has been carried out to investigate the strain–softening phenomena based on the DC model. A representative modified DC (MDC) model, called a “dromedary curve model,” was proposed by Shen [30]. However, describing the constitutive relations when the frozen soil exhibits inconspicuous strain–softening behavior remains a challenge. Recently, new constitutive models have been presented to overcome the drawbacks mentioned above by introducing the statistical continuum damage mechanics theory [31,32,33,34,35], which can better describe the constitutive relations of frozen soil. However, few relevant theoretical investigations have been conducted on the mechanical properties of artificial frozen silty clay subjected to different temperatures, water contents, and confining pressures.

To investigate the mechanical properties of frozen silty clay, an MDC model that describes constitutive relations while considering strain–softening effect was employed. Furthermore, a new statistical damage (SD) constitutive model that regards the failure strain as a random variable was proposed. The parameters employed in these two models are all determined by triaxial experiment results. Finally, the proposed model was further applied to predict the stress–strain curves under some typical experimental conditions performed by other scholars, in order to verify its adaptability. Compared with previous models, this approach is more convenient and easier to apply for engineering design.

## 2. Experimental Conditions

### 2.1. Experimental Material

**Figure 1:**
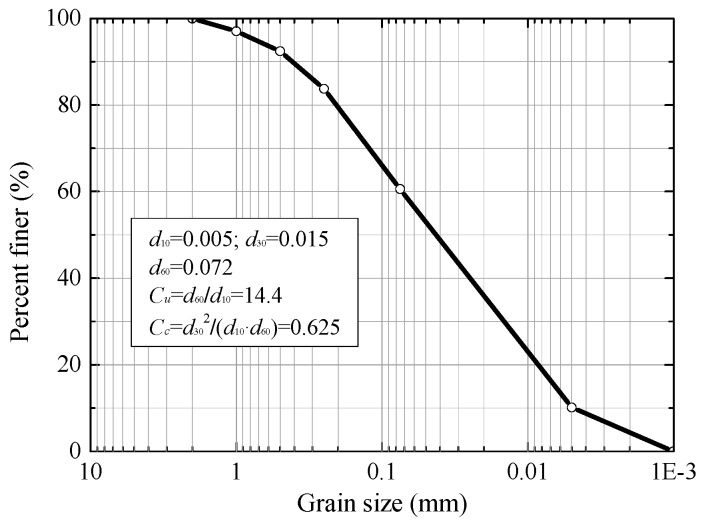
Grain-size distribution of silty clay.

Typical silty clay was collected in Harbin, the grain size distribution of which is shown in Figure 1. The clay’s liquid and plastic limits were 32.8% and 19%, respectively. Testing samples with a dry density of 1640 kg/m^3^ were made from this silty clay.

**Figure 2:**
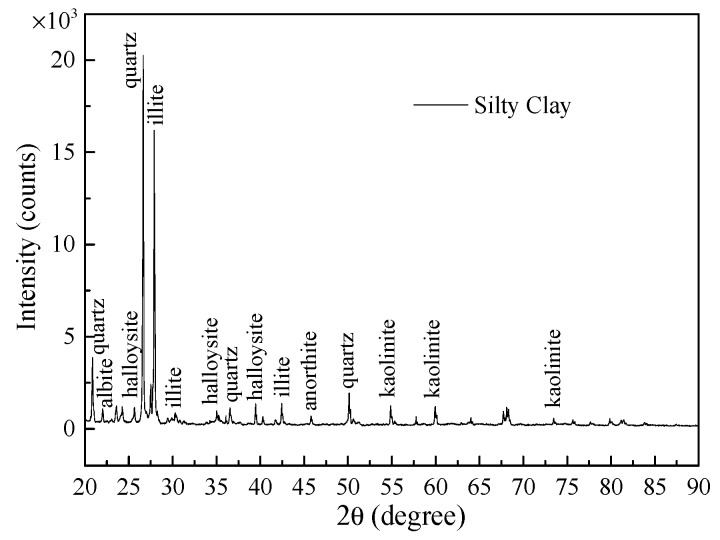
X-ray diffraction spectrum for silty clay.

To make sure the mineralogical characterization of fine-grained soils, X-ray diffractometer (X’PERT, Netherlands) is commonly applied to obtain its spectrum. The sample is passed through a 75-microns IS sieve and the powder sample is oven-dried at 110 °C for 24 h. Soil specimen is placed on the X-ray diffractometer, and the readings are recorded for 2θ angle from 20° to 90°, and at an angular speed of 2°/min with a step size of 0.02. The XRD pattern obtained from the powder samples of silty clay is shown in Figure 2. The major crystalline minerals present in silty clay are kaolinite, illite, halloysite, and quartz from the analysis. And the relative mineral contents of silty clay are shown in Table 1.

### 2.2. Sample Preparation and Test Apparatus 

**Figure 3:**
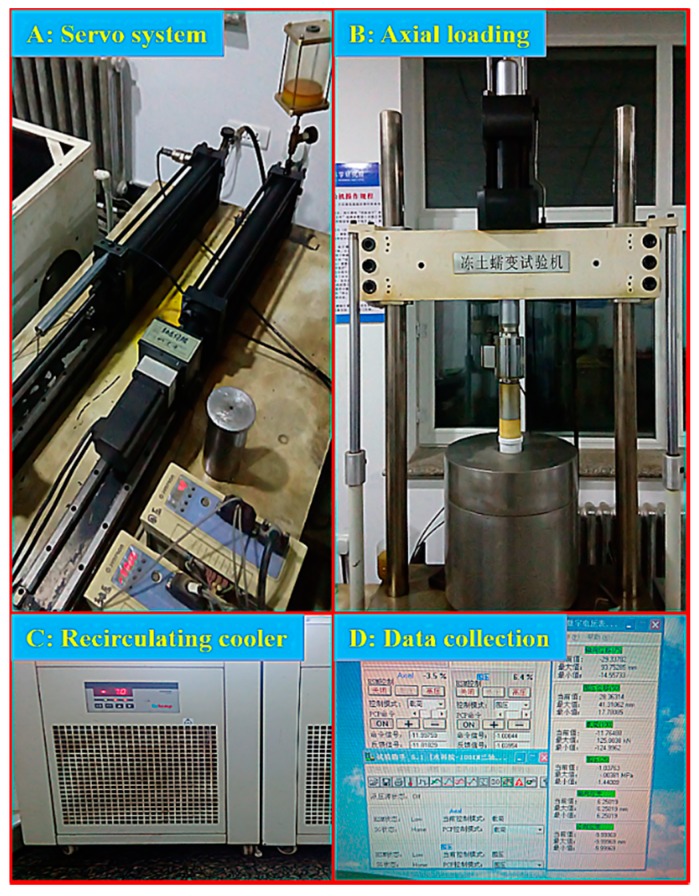
Triaxial shear apparatus for frozen soil.

**Figure 4:**
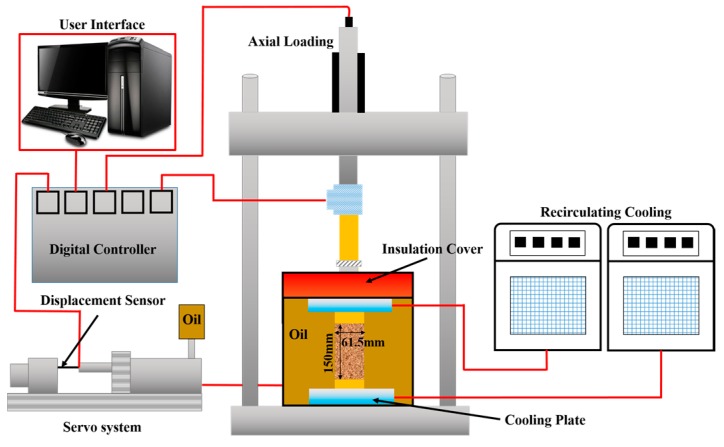
Schematic diagram of the triaxial shear apparatus.

The testing samples were prepared according to the following procedure: (1) the soil was crushed and passed through a 2-mm sieve after drying for 1 day at 115 °C; (2) the target water content was added to the silty clay and sealed for 12 h to ensure that the water content was uniform throughout the samples; (3) the samples were compacted into a three-piece split mold with a diameter of 6.18 cm and a height of 15 cm; (4) the samples were then refrigerated at approximately −35 °C for 48 h to prevent the formation of ice lenses caused by water migration; (5) the three–piece split mold was removed, and the samples were immediately covered with plastic wrap to prevent moisture evaporation; and (6) the samples were preserved in a thermostat for 24 h to achieve the experimental temperature. Twenty-seven effective samples were utilized in triaxial compressive tests to investigate the mechanical properties of frozen silty clay under different temperatures (−5 °C, −10 °C, and −15 °C), confining pressures (0.1, 0.3, and 1.0 MPa) and water contents (17%, 20%, and 23%). The tests were executed in a frozen soil creep apparatus, shown in Figure 3. The schematic diagram of the triaxial shear apparatus is shown in Figure 4. The confining pressure could be adjusted by changing the oil content in the cylinder, ranging from 0 to 25 MPa. The maximum axial force and loading displacement reached 100 kN and 75 mm, respectively. Moreover, a strain-control mode was applied to keep the shear strain rate at 1.2 mm/min.

### 2.3. Experimental Results and Analysis 

**Figure 5:**
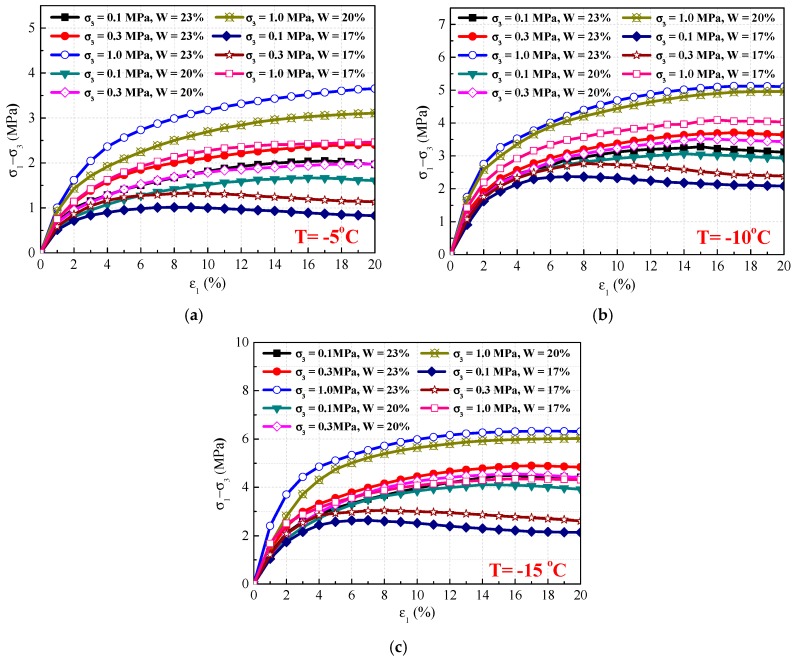
Stress–strain (*σ*_1_–*σ*_3_ and *ε*_1_) curves of frozen silty clay under different temperatures. (**a**) T = −5 °C; (**b**) T = −10 °C; (**c**) T = −15 °C.

Stress–strain curves of the frozen silty clay under different temperatures are shown in Figure 5. Note that the stress–strain curves mainly include three stages: an initial linear elastic stage, a plastic deformation stage, and a strain-softening stage. In the initial elastic stage, the stress–strain curves approached a linear relation. As the axial loading continued, the rate of stress increase became smaller than in the elastic stage, indicating that plastic deformation appeared in the sample. When the strain exceeded 5%, the stress reached more than 60% of the peak strength. With a further increase in strain, strain–softening was observed under lower water contents and confining pressures after reaching a peak strength. However, the stress–strain curves demonstrated a strain–hardening phenomenon under higher water contents, because of cementation effects caused by ice.

**Figure 6:**
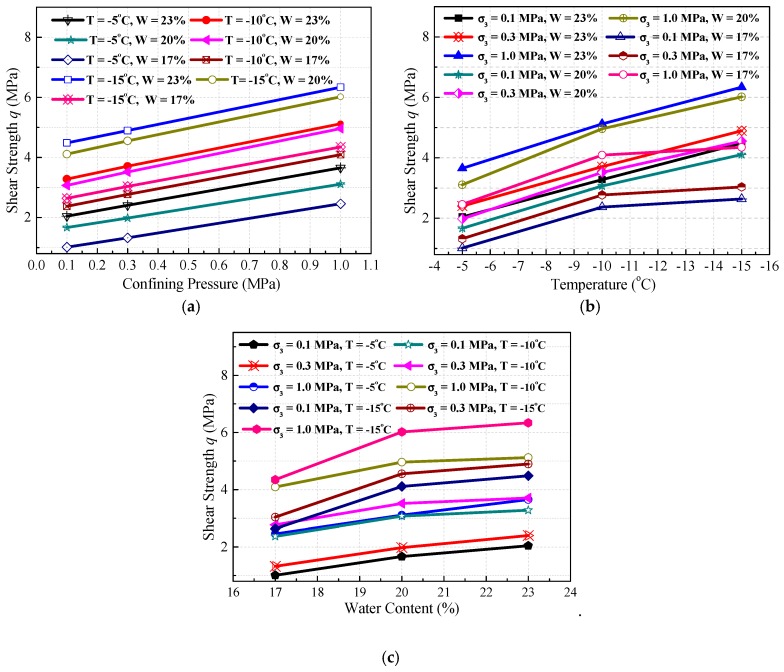
Shear strength varying with confining pressure (**a**), temperature (**b**), and water content (**c**).

Figure 6a illustrates the variation of shear strength (*q*) with confining pressures. As shown in Figure 6a, the shear strength increases linearly with the increase of confining pressure because the increase of confining pressure results in a reduction in the amount of microcracks and an improvement in the integrity of frozen samples. Figure 6b shows the variation of shear strength with temperature. As can be seen, shear strength increases with decreasing temperature, the variation of which can be divided into two stages. The first stage starts from −5 °C to −10 °C, in which the shear strength increases more significantly than that in the second stage. This variation is possibly caused by the changing of ice content, which is less pronounced in the second stage less than that in the first stage because of the unfrozen water content decreases exponentially with the decrease of temperature. The shear strength is determined by the cementation effect of ice inclusions. Figure 6c sketches the variation of shear strength with water content. It can be seen that the shear strength increases with the increase of water content, exhibiting the same variation tendency as seen in Figure 6b. This phenomenon can be explained by the fact that the cementation effect of ice inclusions enhances when the water content increases. However, ice inclusions will become dominant in the frozen soil with a further increase of water content, causing an increase in sliding surface and decreased frictional resistance of soil particles.

## 3. MDC Model for Frozen Silty Clay

### 3.1. Constitutive Model and Parameter Determination

A constitutive model, which can reflect a material’s macroscopic properties, is critical for reasonably determining its mechanical characteristics for geotechnical engineering. The DC model is widely applied in theoretical calculation and practical engineering because some of its parameters have defined physical meaning. Nevertheless, it cannot describe the strain–softening phenomenon in the constitutive model. Herein, an MDC model proposed by Shen [30] was applied to build a constitutive relation of frozen silty clay, expressed as:

(1) $$\sigma_{1}-\sigma_{3}=\frac{\varepsilon_{a}(a+c\cdot \varepsilon_{a})}{{\left(a+b\cdot \varepsilon_{a}\right)}^{2}}$$

where *σ*_1_−*σ*_3_ is deviatoric stress; *ε_a_* denotes axial strain; and *a*, *b,* and *c* are the fitting parameters.

**Figure 7:**
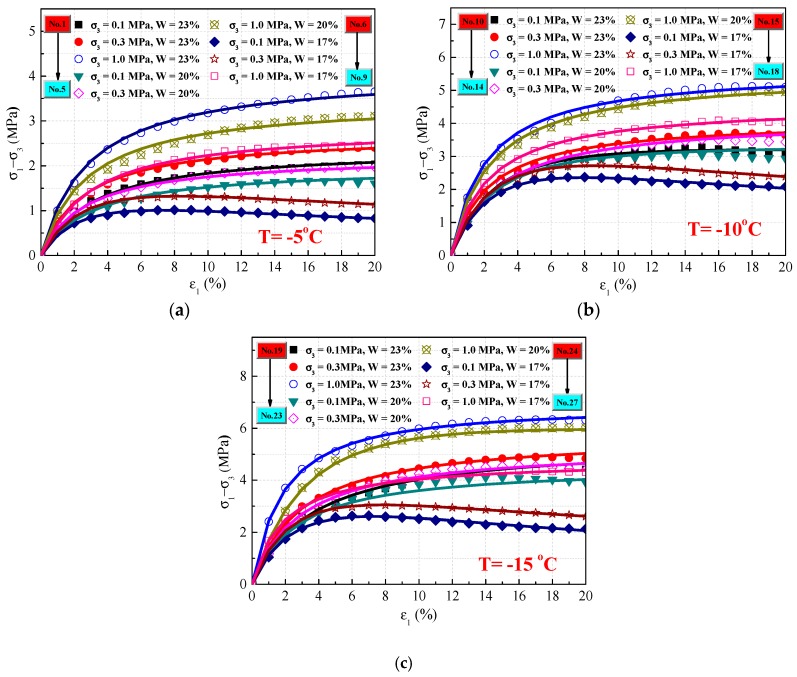
Comparison of the experimental (symbols) and predicted (lines) stress–strain curves of the MDC model. (**a**) T = −5 °C; (**b**) T = −10 °C; (**c**) T = −15 °C.

**Figure 8:**
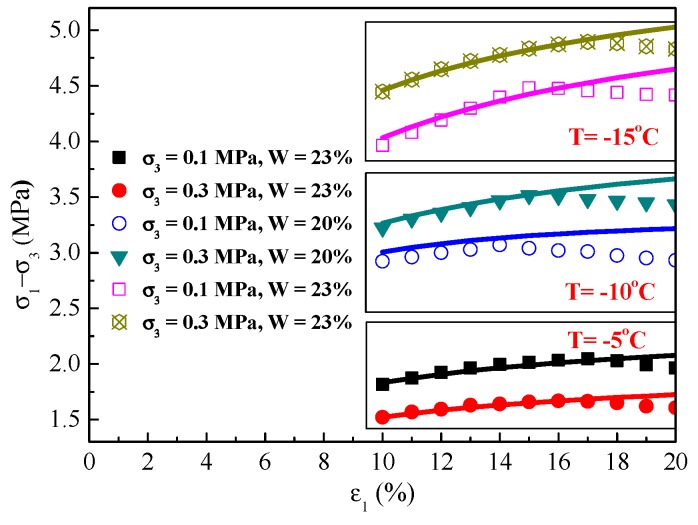
Inset of stress–strain curves between experimental (symbols) and predicted (lines) results of the MDC model.

The fitting parameters and predicted curves of this MDC model under various experimental conditions are shown in Table 2 and Figure 7. The predicted results of the MDC model agree with the reported test results very well under normal conditions. However, these results are not suitable for describing the constitutive relations when the frozen soil exhibits inconspicuous strain-softening, as shown in Figure 8, which is an inset of Figure 7. 

In conventional triaxial compressive tests, *d*σ_2_ = *d*σ_3_ = 0. Therefore, the tangent modulus *E_t_* of frozen soil can be expressed as:

(2) $$E_{t}=\frac{d(\sigma_{1}-\sigma_{3})}{d\varepsilon_{a}}=\frac{a(a+b\cdot \varepsilon_{a})(a-b\cdot \varepsilon_{a}+2c\cdot \varepsilon_{a})}{{\left(a+b\cdot \varepsilon_{a}\right)}^{4}}$$

During the early loading stage, the sample mostly deforms elastically; the initial elastic modulus *E_i_* of frozen soil can be written as follows:

(3) $$E_{i}={\frac{d(\sigma_{1}-\sigma_{3})}{d\varepsilon_{a}}|}_{\varepsilon_{a}\to 0}=\frac{1}{a}$$

The frozen soil’s residual or ultimate strength can be deduced based on Equation (1) as the strain approaches infinity when the frozen soil exhibits strain-softening or strain-hardening behavior, respectively, which can be expressed as:

(4) $${\left(\sigma_{1}-\sigma_{3}\right)|}_{\varepsilon_{a}}{}_{\to \infty }=\frac{c}{b^{2}}$$

Based on triaxial compressive experiment results conducted under different temperatures, confining pressures, and water contents, parameter *a*, which is related to initial elastic modulus *E_i_*, and parameters *b* and *c*, which are related to the residual and ultimate strengths, respectively, can be determined by regressing experimental results.

### 3.2. STRAIN-Softening Criterion of the MDC Model

For strain-softening materials, there exists a maximal stress destruction point on the stress–strain curves, and shear strength can be observed in Figure 5. Then, the initial strain softening is determined by calculating the derivative of the deviatoric stress and setting it to zero:

(5) $$\varepsilon_{rs}=\frac{a}{b-2c}$$

where *ε_rs_* is the initial softening strain.

By substituting Equation (5) into Equation (1), the strain-softening criterion of the MDC model can be expressed as:

(6) $$(b-c\pm \delta_{e})q=0.25$$

where *δ_e_* is a positive error correction term that depends on the experimental results and *q* represents the maximum deviatoric stress corresponding to the initial softening strain.

The predicted model is adequate for a 95% confidence interval, so the length of the confidence interval for parameters *b* and *c* can be described as:

(7) $$\delta_{k}=\frac{2\sigma_{k}}{\sqrt{n}}Z_{0.5\alpha }(k=b,c)$$

where *δ_b_* and *δ_c_* are the lengths of the confidence intervals of parameters *b* and *c*, respectively; *σ_b_* and *σ_c_* denote the standard deviations of *b* and *c*, respectively; *α* represents the significance level; *Z* is a random variable that satisfies the normal distribution; and *n* is the number of samples.

Therefore, the length of the confidence interval of *b*–*c* can be given as follows:

(8) $$\delta_{t}=(\delta_{b}+\delta_{c})=\frac{2(\sigma_{b}+\sigma_{c})}{\sqrt{n}}Z_{0.5\alpha }$$

where *δ_t_* is the length of the confidence interval of *b*–*c*.

When the condition *δ_e_* ≤ 0.5*δ_t_* is satisfied, Equation (8) can serve as the strain-softening criterion for artificially frozen soil. To validate this criterion, the fitting results and experimental values for the strain softening curves are listed in Table 3. Note that *δ_e_* ≤ 0.5*δ_t_* is satisfied when the frozen soil exhibits obvious strain-softening during a triaxial compression experiment. Calculated initial softening strain *ε_rs_* and experimental initial softening strain *ε_re_* are also compared in Table 3, showing that *ε_rs_* is always within the range of *ε_re_*. However, frozen soil with inconspicuous strain-softening behavior cannot be described using this criterion and the *ε_rs_* value obtained by applying this criterion is unreasonable.

## 4. SD Model for Frozen Soil

### 4.1. SD Constitutive Model 

To overcome the limitations of the previous model, an SD model with the failure strain as a random variable, based on the MDC model, was applied. Based on the Lemaître principle of equivalence strain [36], strain *ε_a_* on the damaged material caused by macroscopic stress *σ* is equivalent to strain *ε_b_* on the undamaged material caused by effective stress *σʹ*. Hence, according to the MDC model, the damage constitutive model for frozen soil can be expressed as:

(9) $$\sigma =\sigma^{\prime }(1-D)$$

(10) $$\sigma_{1}{}^{\prime }-\sigma_{3}{}^{\prime }=\frac{\varepsilon_{b}(a+c\cdot \varepsilon_{b})}{{\left(a+b\cdot \varepsilon_{b}\right)}^{2}}$$

where *D* is a damage variable within the range 0–1. Then, substituting Equation (9) into Equation (10), the damage constitutive model based on MDC can be expressed as:

(11) $$\sigma_{1}-\sigma_{3}=\frac{\varepsilon_{a}(a+c\cdot \varepsilon_{a})}{{\left(a+b\cdot \varepsilon_{a}\right)}^{2}}(1-D)$$

The SD theory is a powerful tool for describing the stress–strain relations of frozen soil caused by the random distribution of fissures and cavities. The shear strength and axial strain are always regarded as random variables in an SD model. Li [31] pointed out that using the failure strain as a random variable is somewhat unreasonable, as its physical concept is ambiguous in classical plastic theory. However, the failure strain as a random variable is more simply applied without detriment to accuracy when establishing a constitutive model.

The normal, lognormal, and Weibull distributions are mainstream approaches to fitting probabilistic distributions of failure strain in frozen soil under various experimental conditions. Lai [34] reported that a Weibull distribution can better describe the probabilistic distribution of failure strain than other methods; the probability density function of a Weibull distribution can be obtained as follows:

(12) $$f(\varepsilon_{a})=\frac{m}{F_{0}}{\left(\frac{\varepsilon_{a}}{F_{0}}\right)}^{m-1}\exp\left[-{\left(\frac{\varepsilon_{a}}{F_{0}}\right)}^{m}\right]$$

where *m* and *F*_0_ are Weibull parameters.

When the strain reaches a certain level, the number of damaged elements can be expressed as

(13) $$N(\varepsilon_{a})=\int_{0}^{\varepsilon_{a}}Nf(x)dx=N\left\{1-\exp\left[-{\left(\frac{\varepsilon_{a}}{F_{0}}\right)}^{m}\right]\right\}$$

where *N* denotes the number of all elements and *N* (*ε_a_*) is the number of damaged elements.

Here, damage variable *D* in the SD mechanics needs to be redefined as:

(14) $$D=\frac{N(\varepsilon_{a})}{N}$$

Substituting Equation (13) into Equation (14), the damage variable *D* can be rewritten as:

(15) $$D(\varepsilon_{a})=1-\exp\left[-{\left(\frac{\varepsilon_{a}}{F_{0}}\right)}^{m}\right]$$

Ultimately, by substituting Equation (15) into Equation (11), the SD constitutive model can be obtained as follows:

(16) $$\sigma_{1}-\sigma_{3}=\frac{\varepsilon_{a}(a+c\cdot \varepsilon_{a})}{{\left(a+b\cdot \varepsilon_{a}\right)}^{2}}\exp\left[-{\left(\frac{\varepsilon_{a}}{F_{0}}\right)}^{m}\right]$$

**Figure 9:**
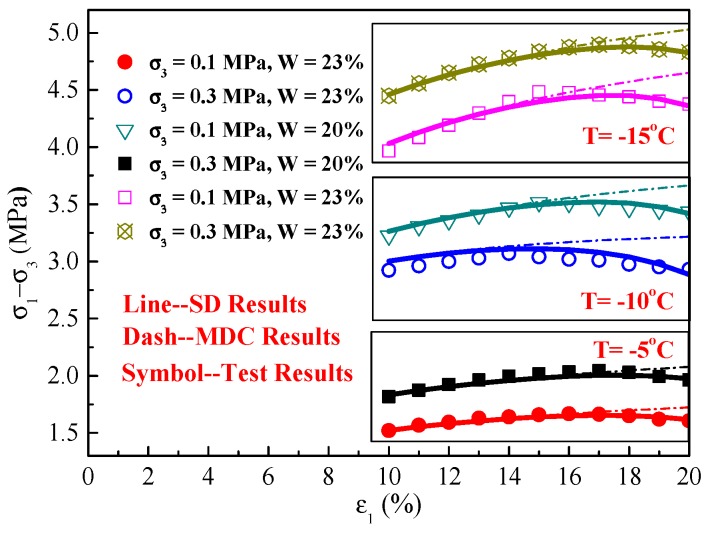
Comparison of experimental and predicted stress–strain curve results.

Compared with the SD and MDC models, the initial elastic model should be consistent and parameters *b* and *c* should be the same as in these two models for the sake of convenient transformation. To validate the SD constitutive model, typical experimental results with inconspicuous strain-softening behavior were applied. The fitting parameters are shown in Table 4. Moreover, the stress–strain curves from the experimental and predicted results are compared in Figure 9. Besides, the correlation coefficient of the SD model has a slight improvement compared with the previous model, which means that the SD model was considerably more accurate in describing inconspicuous strain-softening behavior in frozen soil than the MDC model.

In the SD model, *m* and *F*_0_ are scale and shape parameters, respectively, dependent on the position of the initial softening strain. The parameters *m* and *F*_0_ can be calculated using a nonlinear regression method, from the experimental results of the triaxial compression. In practice, many problems can be attributed to the optimization of nonlinear least squares. Herein, the Gauss–Newton algorithm is applied to estimate the parameters *m* and *F*_0_. It is based on the implemented first derivatives of the components of the vector function. In special cases it can give quadratic convergence as the Newton method does for general optimization.

For any choice of **x** = [*m*, *F*_0_], the residuals can be computed:

(17) $$f_{i}(x)={\left(\sigma_{1}-\sigma_{3}\right)}_{i}-A\exp\left[-{\left(\frac{\varepsilon_{a}}{F_{0}}\right)}^{m}\right]$$

where parameter *A* is the predicted result by the MDC model.

The residuals *f_i_*(**x**) in this method can be replaced by using first-order approximation of Taylor expansion.

(18) $$g_{i}(x)=f_{i}(x^{k})+\nabla f_{i}{\left(x^{k}\right)}^{T}(x-x^{k})$$

The objective function *F*(**x**) is a quadratic sum of several functions *f_i_*(**x**), which can be formulated as:

(19) $$F(x)=\sum_{i=1}^{m}g_{i}{}^{2}(x)=(J_{k}x-b{)}^{T}(J_{k}x-b)$$

(20) $$J_{k}={\left[\nabla f_{1}{\left(x^{k}\right)}^{T},\nabla f_{2}{\left(x^{k}\right)}^{T},\nabla f_{3}{\left(x^{k}\right)}^{T},\ldots ,\nabla f_{m}{\left(x^{k}\right)}^{T}\right]}^{T}$$

(21) $$b={\left[\nabla f_{1}{\left(x^{k}\right)}^{T}x^{k}-f_{1}(x^{k}),\nabla f_{2}{\left(x^{k}\right)}^{T}x^{k}f_{2}(x^{k}),\ldots ,\nabla f_{m}{\left(x^{k}\right)}^{T}x^{k}f_{m}(x^{k})\right]}^{T}$$

(22) $$f_{k}={\left[f_{1}(x^{k}),f_{2}(x^{k}),f_{3}(x^{k}),\ldots ,f_{m}(x^{k})\right]}^{T}$$

By calculating the derivative of Equation (19) and setting it to zero, the iteration form of the Gauss–Newton method can be expressed as:

(23) $$x-x^{k}=-{\left(J_{k}{}^{T}J_{k}\right)}^{-1}J_{k}{}^{T}f^{k}$$

Considering a set of experimental data points, (*ε_a_*_1_, (σ_1_-σ_3_)_1_), (*ε_a_*_2_, (σ_1_-σ_3_)_2_), …, (*ε_a_*_n_, (σ_1_-σ_3_)_n_), the parameters *m* and *F*_0_ can be obtained using the following procedure:

- Set the initial value of **x** = [*m*, *F*_0_] and threshold ε;
- Calculate *f_i_*(**x**) and obtain the vector *f_k_*;
- Calculate *f_i_^’^*(**x**) and obtain the Jacobian matrix *J_k_*;
- Calculate **x**^k+1^ by using the iteration form of the Gauss-Newton method;
- Judge the condition ‖**x**^*k*+1^ − **x**^*k*^‖ < *ε*, if it is not satisfied, repeat step (2) until the condition is satisfied.

After the parameters *m* and *F*_0_ are obtained under different experimental conditions, an empirical formula for the particular experimental sample can be adopted to quantify the relationship between the two parameters (*m* and *F*_0_) and three variables (temperature, water content, and confining pressure).

### 4.2. Strain-Softening Criterion of the SD Model

The initial softening strain is obtained by calculating the derivative of the deviatoric stress in Equation (16) and setting it to zero, as in Section 3.2.

(24) $$\frac{d(\sigma_{1}-\sigma_{3})}{d\varepsilon_{a}}=a(a-b\cdot \varepsilon_{a}+2c\cdot \varepsilon_{a})-\varepsilon_{a}(a+c\cdot \varepsilon_{a})(a+b\cdot \varepsilon_{a})\cdot \frac{m}{F_{0}}{\left(\frac{\varepsilon_{a}}{F_{0}}\right)}^{m-1}=0$$

Here, reference strain *ε_rep_* is introduced and assigned a value of 17 because the SD model is used to describe inconspicuous strain-softening behavior. The reference strain can also be assigned other values, as long as the ratio of strain to reference strain is close to 1. In this experiment, because the interval of the initial softening strain is between 15 and 18, the ratio of strain to reference strain can be controlled within a range of 0.88 to 1.05.

(25) $$bc\varepsilon_{a}{}^{2}+a(b+c)\cdot \varepsilon_{a}-\frac{F_{0}}{m}\cdot {\left(\frac{F_{0}}{\varepsilon_{a}}\right)}^{m-1}\left(\frac{a^{2}}{\varepsilon_{rep}}-ab+2ac\right)+a^{2}=0$$

By setting *C* = −*a* (*b* + *c*) and *D* = *bc*, Equation (25) can be solved as

(26) $$2D\varepsilon_{a}-C=\sqrt{K\varepsilon_{a}{}^{1-m}+E}$$

(27) $$K=4D{\left(\frac{1}{F_{0}}\right)}^{1-m}\frac{F_{0}}{m}\left(\frac{a^{2}}{\varepsilon_{rep}}-ab+2ac\right)$$

(28) $$E=C^{2}-4Da^{2}$$

Because parameter *E* is far smaller than *K*, it can be ignored. By taking the log of both sides of Equation (26) and introducing the reference strain again, the initial softening strain can be obtained as:

(29) $$\varepsilon_{rs}=e^{\frac{\ln K-2\ln\left(2D-\frac{C}{\varepsilon_{rep}}\right)}{\left(1+m\right)}}$$

The calculated and experimental initial softening strains are also shown in Table 4. The calculated initial softening strain is always within the range of the experimental results and there is a good agreement between the experimental and predicted results of the SD model, except for a small bias in one test case. This bias is possibly caused by the reference strain being assigned a value of 17, which can produce errors under certain experimental conditions. Overall, only an approximation method to predict the initial softening strain is provided in this paper.

### 4.3. Model Verification and Analysis

**Figure 10:**
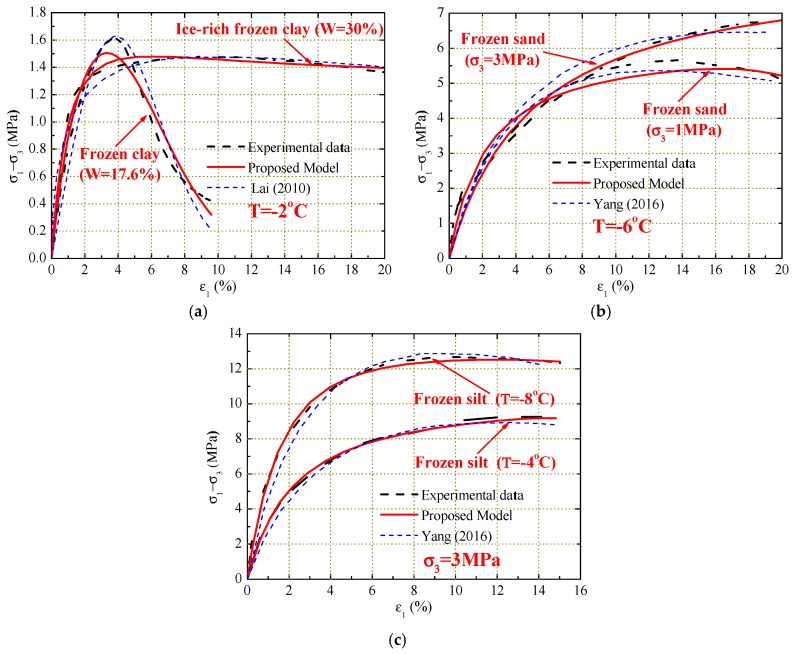
Comparison of the experimental and predicted stress–strain curves. (**a**) water content; (**b**) confining pressure; (**c**) temperature.

In order to verify the feasibility and adaptability of the model, comparisons of stress–strain curves between experimental results obtained by other researchers and predicted results in this study are performed. According to the experimental results in the studies by Lai [5] and Yang [37], the unknown parameters need to be determined first and then the procedure should be applied to predict the stress–strain curves for some typical experimental conditions. Figure 10 displays the comparisons of experimental and predicted stress–strain curves for frozen clay, sand, and silt. It can be seen that the procedure gives relatively good prediction of the experimental stress–strain curves for various frozen soils under different water contents, confining pressures, and temperatures. Although there is a large difference between experimental and predicted results at the condition of water content of 17.6% compared with the model proposed by Lai [5], other predictions are slightly better and more convenient than the original models. Particularly, when the stress reaches its peak point, the experimental and predicted curves overlap. The mechanical properties of frozen soil under different water contents, confining pressures, and temperatures are found to be correctly described by the proposed model.

## 5. Conclusions

A series of triaxial compression tests were conducted to investigate the mechanical properties of the frozen silty clay at –5 °C, –10 °C, and –15 °C, under different confining pressures and water contents. An MDC model that considers strain-softening effects was further applied to predict the triaxial strength and deformation characteristics of frozen silty clay. Finally, beginning with SD theory, a constitutive model of frozen soil under triaxial compression was established and confirmed by experimental outcomes. Based on this study, the following conclusions can be drawn:

(1) The experimental results show that the mechanical properties of frozen silty clay are very sensitive to temperature, confining pressure, and water content. The stress–strain curves exhibit strain-softening under lower confining pressures and water contents. Moreover, frozen silty clay under lower temperatures and higher confining pressures and water contents shows greater shear strength in triaxial compression experiments.

(2) The MDC model is employed to describe the constitutive relation of frozen silty clay, while considering strain–softening effects. The results predicted by the MDC model are in agreement with the experimental results under conditions of strain-hardening and clear strain-softening. Furthermore, the strain–softening criterion of the MDC model was presented and found that the calculated initial softening strain is always within the range of the experimental initial softening strain when the frozen soil exhibits conspicuous strain–softening behavior.

(3) In this paper, an SD constitutive model was proposed with the failure strain as a random variable. Compared with results predicted by the MDC model, the agreement between prediction results from the SD and test results leave room for improvement, particularly for cases of inconspicuous softening. A softening-strain criterion based on the SD model was also introduced and it was found that the calculated initial softening strain is always within the range of the experimental initial softening strain when the frozen soil exhibits inconspicuous strain–softening behavior. 

(4) The comparison of stress–strain curves between the experimental results by other researchers and results calculated in this study showed that the proposed model can correctly predict the deformation behavior. Moreover, the model is more convenient and easier to apply for engineering design, and provides a good reference for predicting the mechanical properties of frozen soil.

## Figures and Tables

**Table 1:** Relative mineral contents of silty clay.

Mineral Composition	Quartz	Illite	Halloysite	Kaolinite	Albite	Anorthite	Unknown
Relative content (%)	39.9	10.2	14.7	5.6	13.2	12.4	4.0

**Table 2:** Computational parameters of the modified Duncan–Chang (MDC) model.

No.	Fitting Parameters				No.	Fitting Parameters			
	a	b	c	R^2^		a	b	c	R^2^
1	1.292	0.416	0.415	0.9885	15	0.421	0.191	0.191	0.9972
2	0.960	0.371	0.372	0.9939	16	0.756	0.120	0.011	0.9942
3	0.690	0.244	0.244	0.9988	17	0.709	0.101	0.009	0.9976
4	1.537	0.502	0.501	0.9898	18	0.486	0.238	0.260	0.9989
5	1.124	0.448	0.447	0.9970	19	0.660	0.191	0.189	0.9943
6	0.796	0.288	0.288	0.9911	20	0.507	0.174	0.174	0.9976
7	1.662	0.265	0.017	0.9919	21	0.279	0.097	0.065	0.9988
8	1.440	0.204	0.015	0.9859	22	0.636	0.314	0.450	0.9898
9	1.139	0.565	0.914	0.9983	23	0.545	0.198	0.198	0.9907
10	0.707	0.123	0.045	0.9956	24	0.481	0.152	0.152	0.9919
11	0.534	0.265	0.278	0.9963	25	0.681	0.098	0.002	0.9995
12	0.368	0.179	0.181	0.9946	26	0.580	0.090	0.008	0.9983
13	0.727	0.143	0.066	0.9974	27	0.386	0.209	0.209	0.9959
14	0.669	0.239	0.239	0.9973	–	–	–	–	–

**Table 3:** Criterion for strain-softening curves.

No.	q(b–c)	δ_b_	δ_c_	0.5δ_t_	δ_e_	ε_rs_ (%)	ε_re_ (%)
7	0.2514	0.0346	0.0284	0.0315	0.0014	7.20	7~9
8	0.2504	0.0322	0.0266	0.0294	0.0004	8.28	8~10
16	0.2514	0.0098	0.0080	0.0089	0.0014	7.95	6~8
17	0.2550	0.0092	0.0078	0.0087	0.0050	8.54	7~9
25	0.2533	0.0074	0.0058	0.0071	0.0033	6.75	6~8
26	0.2492	0.0064	0.0054	0.0059	0.0008	7.84	7~9

**Table 4:** Computational parameters of the statistical damage (SD) model.

	Fitting Parameters							
	a	b	c	F_0_	m	ε_rs_ (%)	ε_re_ (%)	R^2^
1	1.292	0.416	0.415	28.61	8.322	17.63	16~18	0.9916
4	1.537	0.502	0.501	28.20	8.012	17.17	16~18	0.9952
13	0.727	0.143	0.067	27.12	7.306	14.61	14~16	0.9986
14	0.669	0.239	0.239	27.86	7.967	16.81	15~17	0.9988
19	0.660	0.182	0.181	28.01	8.043	17.36	15~17	0.9961
20	0.507	0.174	0.174	28.91	8.643	17.92	16~18	0.9982

## Notation List

**Symbol**	**Description**
σ_1_−σ_3_	Deviatoric stress
ε_a_	Axial strain
a	Parameter related to initial elastic modulus
b	Parameter related to residual and ultimate strengths
c	Parameter related to residual and ultimate strengths
E_t_	Tangent modulus
E_i_	Initial elastic modulus
ε_rs_	Calculated initial softening strain
q	Shear strength
δ_e_	Positive error correction term
δ_b_	Length of the confidence interval for parameter b
δ_c_	Length of the confidence interval for parameter c
α	Significance level
Z	Random variable that satisfies normal distribution
n	Number of samples
σ_b_	Standard deviation of parameter b
σ_c_	Standard deviation of parameter c
δ_t_	Length of the confidence interval of b-c
ε_re_	Experimental initial softening strain
σ_1_ʹ-σ_3_ʹ	Effective deviatoric stress
D	Damage variable
N	Total number of elements
N(ε_a_)	Number of damaged elements
f(ε_a_)	Probability density function of the Weibull distribution
m	Scale parameter of the Weibull distribution
F_0_	Shape parameter of the Weibull distribution
f_i_(**x**)	Residuals between test and simulation results
x	Parameter set
x^k^	Parameter value at k-th iteration
A	Predicted results calculated by the MDC model
g_i_(**x**)	First order Taylor expansion of f_i_(**x**)
F(**x**)	Objective function
J_k_	Jacobian matrix
ε	Error threshold
ε_rep_	Reference strain
